# Exosomes from neuronal stem cells may protect the heart from ischaemia/reperfusion injury via JAK1/2 and gp130

**DOI:** 10.1111/jcmm.16515

**Published:** 2021-04-01

**Authors:** Miroslava Katsur, Zhenhe He, Vladimir Vinokur, Randolph Corteling, Derek M Yellon, Sean M Davidson

**Affiliations:** ^1^ The Hatter Cardiovascular Institute University College London London UK; ^2^ Department of Biochemistry and Molecular Biology Institute of Medical Research Israel‐Canada The Hebrew University of Jerusalem Jerusalem Israel; ^3^ ReNeuron Ltd Pencoed, Bridgend UK

**Keywords:** cardiac, exosomes, extracellular vesicles, infarction, ischaemia and reperfusion, mitochondria, neuronal stem cells

## Abstract

Myocardial infarction requires urgent reperfusion to salvage viable heart tissue. However, reperfusion increases infarct size further by promoting mitochondrial damage in cardiomyocytes. Exosomes from a wide range of different cell sources have been shown to activate cardioprotective pathways in cardiomyocytes, thereby reducing infarct size. Yet, it is currently challenging to obtain highly pure exosomes in quantities enough for clinical studies. To overcome this problem, we used exosomes isolated from CTX0E03 neuronal stem cells, which are genetically stable, conditionally inducible and can be produced on an industrial scale. However, it is unknown whether exosomes from neuronal stem cells may reduce cardiac ischaemia/reperfusion injury. In this study, we demonstrate that exosomes from differentiating CTX0E03 cells can reduce infarct size in mice. In an in vitro assay, these exosomes delayed cardiomyocyte mitochondrial permeability transition pore opening, which is responsible for cardiomyocyte death after reperfusion. The mechanism of MPTP inhibition was via gp130 signalling and the downstream JAK/STAT pathway. Our results support previous findings that exosomes from non‐cardiomyocyte‐related cells produce exosomes capable of protecting cardiomyocytes from myocardial infarction. We anticipate our findings may encourage scientists to use exosomes obtained from reproducible clinical‐grade stocks of cells for their ischaemia/reperfusion studies.

## 1 INTRODUCTION

Acute myocardial infarction is typically caused by a coronary artery blockage.^1^ Timely reperfusion is necessary to salvage ischaemic myocardium. However, reperfusion itself causes a degree of injury and is responsible for ~50% cell death caused by ischaemia and reperfusion (IR) in the heart.^2^ Upon reoxygenation of cardiomyocytes, electron flow returns to the electron transport chain but electrons are initially transferred through complex I in reverse, generating reactive oxygen species (ROS).^3^ Excess mitochondrial ROS and calcium result in opening of mitochondrial permeability transition pore (mPTP), which causes cardiomyocyte death. Therefore, prevention of mitochondrial injury is one important aspect of therapies aiming to reduce infarct size.^4^ mPTP opening can be prevented by direct chemical inhibition of the regulatory protein cyclophilin D,^5^ or by certain protein kinases that have been shown to limit mPTP formation.^6^, ^7^ These pro‐survival kinases, such as PI3K/AKT and JAK/STAT, are defined as the reperfusion injury salvage kinase (RISK) pathway and the survivor activating factor enhancement pathway, respectively.^8^, ^9^ However, to date, no treatment specifically targeting IR injury has been successfully translated from laboratory to patients (reviewed in ^10^).

There is currently great interest in the potential for cardioprotection by exosomes. Exosomes are nano‐sized, extracellular vesicles that are able to signal between cells. Exosomes from a range of different types of stem cells have been shown to reduce infarct size caused by cardiac IR injury, including those produced by mesenchymal stem cells (MSC), cardiac progenitor cells, embryonic stem cells, W8B2+ stem cells, amniotic fluid stem cells and bone‐marrow‐derived stem cells.^11^, ^12^, ^13^, ^14^ Furthermore, cardioprotection can also be seen with exosomes purified from non‐stem‐cell origins such as endothelial cells, dendritic cells, adipose stem cells and blood plasma.^13^, ^15^, ^16^, ^17^ Importantly, many types of exosomes have been shown to activate kinases of the RISK pathway.^15^, ^18^, ^19^, ^20^, ^21^ These data suggest that the specific source of exosomes is not a major factor in determining their ability to protect the heart, so long as they activate cardioprotective signalling pathways. On the other hand, some cell culture or growth conditions can affect exosome activity. For example, type II diabetes and hyperglycaemia can prevent exosomes from being able to protect the heart.^22^, ^23^

Given the clinical need for an effective treatment for cardiac IR injury, and the fact that cardioprotection has been seen using exosomes from various cell‐type including non‐cardiac cells, we decided to investigate whether cardioprotection could be achieved using a clinical‐grade preparation of exosomes obtained from a human, neural stem cell line called CTX0E03. Despite being neural, exosomes from these cells were attractive for a number of reasons. First, the exosome‐producing stem cells are grown in a high‐yield, GMP‐grade production facility, offering the potential for future expansion for routine production of GMP‐grade exosomes that can be directly used in patient studies. Second, the CTX0E03 cell line is genetically stable, conditionally immortalized and conditionally proliferating,^24^ which facilitates the isolation of exosomes from either proliferating or differentiated cells (referred to as ExoPr0 and ExoDiff, respectively)—this is useful for the comparison of exosomes from different conditions. Third, the sequential isolation procedure we developed, consisting of tangential flow filtration followed by size exclusion chromatography, is expected to result in highly purified exosomes. Furthermore, during this entire isolation process, the exosomes remain in solution in a standard physiological buffer, which avoids the use of harsh and potentially damaging techniques such as ultracentrifugation or precipitation. The combination of these techniques can potentially achieve high exosome purity by removing particles larger or smaller than exosomes, respectively, without compromising exosome functional properties.^25^

CTX0E03 cells, themselves, have previously been demonstrated to be beneficial in IR injury in the setting of ischaemic stroke.^26^, ^27^ Interestingly, CTX0E03 cells were also shown to induce angiogenesis in mouse models of hind limb ischaemia, which involves activation of the same pro‐survival RISK pathway known to be involved in cardioprotection.^11^ The mechanism of action of the CTX0E03 stem cells is believed to involve the transfer of exosomes to the injured cells.^28^, ^29^

We, therefore, hypothesized that ExoPr0 and ExoDiff would protect cardiomyocytes from cell death by activating cardioprotective pathways that protect mitochondria, thereby limiting infarct size following ischaemia and reperfusion. We first characterized the exosomes to determine their size, appearance and expression of protein exosomal markers, in accordance with recommendations by minimal information for studies of extracellular vesicles 2018 (MISEV2018).^25^ We then evaluated the potential of ExoDiff and ExoPr0 exosomes to reduce cardiac ischaemia/reperfusion injury in vivo and delay mPTP opening caused by ROS in vitro. We also investigated the mechanism of protection by using inhibitors of the RISK pathway and other cardioprotective kinases.

## 2 MATERIALS AND METHODS

### 2.1 Exosome isolation

Small extracellular vesicles (hereafter: exosomes) from differentiating (ExoDiff) and proliferating (ExoPr0) CTX0E03 human neuronal stem cell (hNSC) cultures were isolated from tissue culture medium conditioned by CTX0E03 cells, using a previously described, sequential method of tangential flow filtration followed by size exclusion chromatography.^28^, ^29^, ^30^ In brief, cells were cultured using either T flasks (ExoPr0), as described previously,^31^ or Integra CELLine bioreactors (ExoDiff) in a chemically defined growth media (Dulbecco's Modified Eagles Medium:F12 medium (Invitrogen) (DMEM:F12), supplemented with human albumin solution (0.03%; Grifols), L‐glutamine (2 mmol/L; Gibco), human transferrin (5 μg/mL; Sigma), putrescine dihydrochloride (16.2 μg/mL; Sigma), human insulin (5 μg/mL; Sigma), progesterone (60 ng/mL; Sigma), sodium selenite (40 ng/mL; Sigma), epidermal growth factor (20 ng/mL; Sigma) and basic fibroblast growth factor (10 ng/mL; Invitrogen). ExoPro conditioned media (CM) was collected from 80% to 90% confluent hNSC in sterile conditions, and filtered using a filter unit (Millipore, SCGPU05RE) with a 0.22 μm membrane to remove intact cells and cell debris.^31^ To allow the cells to partially differentiate in the case of ExoDiff, Integra CELLine cultures were allowed to grow for between 6 and 15 weeks prior to harvesting the CM. The CM for both ExoPr0 and ExoDiff were then concentrated, and buffer changed by diafiltration into PBS using a 100 kDa molecular weight cut‐off hollow‐fibre polyethersulfone (mPES) (Repligen, S02‐E100‐05‐N) membrane followed by size exclusion chromatography (IZON, qEV) to eliminate non‐vesicular material following the manufactures recommendations.

### 2.2 Nanoparticle tracking analysis

Particle quantity and size in exosome samples were determined by nanoparticle tracking analysis using a NanoSight LM10‐HS instrument with a 488nm laser unit, syringe pump and charge‐coupled camera (Malvern Panalytical).^32^ Dilutions of ExoDiff or ExoPr0 in water were injected at speed 20 and illuminated with the laser. The scattered light was recorded as three videos of 90sec. The detection level was set at 5. NanoSight software (v.3.1) was used for data analysis.

### 2.3 Spectrophotometry

Exosome samples or bovine serum albumin (BSA) diluted in water were loaded on a low‐volume microplate LVis (BMG Labtech). Protein absorbance was read at 280nm with a FLUOstar plate reader (BMG Labtech). Protein concentration in ExoDiff and ExoPr0 was calculated using the equation of the BSA standard curve.^33^

### 2.4 Dissociation‐enhanced lanthanide fluorescence immunoassay (DELFIA)

Exosome markers were detected with DELFIA.^34^ Specifically, ExoDiff or ExoPr0 dilutions or their diluent, phosphate buffer saline (PBS), were loaded in duplicates on high‐binding 96‐well plates (R&D Systems, DY990) and incubated at 4°C. Next day, the plates were washed three times with DELFIA washing buffer before any reagent was added to wells (PerkinElmer, 1244‐114). First, plates were blocked with 1% BSA in PBS for 1 h at room temperature. Then, plates were incubated with anti‐CD9 (1:500), CD63 (1:200) or CD81 (1:500; BD Biosciences, 555 370, 556 019, 555 675). After 2 h, plates were incubated with appropriate secondary antibodies (1:2,000; Abcam, ab98691, ab97073) for 1 h. Afterwards, streptavidin‐europium conjugate in assay buffer (1:1,000) was added to wells for 1 h (PerkinElmer, 1244‐106 and 1244‐30). Finally, plates were washed six times and incubated with enhancement solution, while being shaken at 300rpm for 10 min (PerkinElmer, 1244‐104). The samples were analysed with a PHERAstar plate reader with the following settings: 337 nm excitation, 620 nm detection, 200 μs integration time and 60 μs lag time (BMG Labtech). Fluorescence value of each control, that is PBS incubated with both primary and secondary antibodies was subtracted from sample fluorescence.

### 2.5 Single particle interferometric reflectance imaging (SP‐IRIS)

The presence of exosome markers in ExoDiff and ExoPr0 was confirmed by using ExoView Tetraspanin SP‐IRIS chips (NanoView Diagnostics).^35^ The samples were diluted with 0.05% Tween20 in PBS. Dilutions (35 μL) were added in triplicates on the chips pre‐coated with mouse anti‐CD9, CD63 and CD81 antibodies (NanoView Diagnostics). Mouse IgG was used as a negative control. The chips were incubated at room temperature for 16 h. After three washes with PBS, CD9/Alexa488, CD63/Alexa647 and CD81/Alexa555 secondary antibodies were added on the chips for 2 h (NanoView Diagnostics). Then, the chips were washed once with 0.05% Tween20 in PBS, three times with PBS, and once with water. After drying chips with blotting paper, interferometric images of the chip were acquired with an ExoView reader using the ExoScan software. Fluorescence was quantified with ExoViewer software with sizing threshold set to 50‐200 nm. Then, mean fluorescence of sample replicates, particle size, number and subtype was determined.

### 2.6 Transmission electron microscopy

Exosome samples were imaged by Joel 1010 electron microscope (Joel Ltd.).^36^ Formvar‐carbon coated grids stayed on sample drops at room temperature overnight (Agar Scientific, S138A6). Next day, grids were washed on PBS, contrasted with 50 μL uranyl‐oxalate for 30 s and then dried with blotting paper (Thomas Scientific, C993L46). Afterwards, grids were air‐dried for 5‐10 min and imaged at 80 kV. Immunostaining of CD63 was performed on whole‐mounted exosomes followed by a gold‐labelled goat anti‐mouse IgG secondary antibody, followed by post‐fixation and staining.^36^

### 2.7 Lipid assay

The lipid content of exosomes was determined using the improved, high‐sensitivity 96‐well plate format lipid quantification assay, developed by Visnovitz et al ^37^ for experiments with EVs. In brief, 1.2‐Dioleoyl‐sn‐glycero‐3‐phosphocholine (DOPC) liposomes were prepared as described ^37^ and used to construct a standard curve. 200 µL of 96% sulphuric acid was added to 40 µL standards or exosome samples in in 1.5 mL test tubes (Safe‐Lock tubes, 1.5 ml, 0030 120‐086, Eppendorf AG, Germany). After drying, 120 µL of phospho‐vanillin reagent (50 mg vanillin in 50 mL of 17% phosphoric acid) was added to each tube and vortexed. Next, 280 µL of each sample was transferred to a 96‐well plate and the colour reaction was allowed to develop for 1 h at 37°C. Absorbance at 540 nm was determined with a fluostar plate reader (BMG Labtech), and lipid content determined with reference to the standard curve.

### 2.8 Ethical approval

All animals received humane care in accordance with the Animal Welfare and Ethical Review Body, and in vivo work was conducted according to the UK Home Office Guide on the Operation of Animals (Scientific Procedures) Act 1986, under Project Licence number PPL 70/8556. The experiments were performed according to the guidelines from Directive 2010/63/EU of the European Parliament on the Protection of animals used for scientific purposes or the National Institutes of Health guidelines.

### 2.9 In vivo ischaemia‐reperfusion injury

Male wild‐type C57Bl/6 mice (12‐ to 15‐week‐old) were anaesthetized intraperitoneally with 80 mg/kg pentobarbital. They were placed in supine position on a heating pad (36.5‐37.5°C). After tracheostomy, artificial ventilation was established using a 19G cannula connected to a MiniVent type 845 animal ventilator (Harvard Apparatus). Flow rate was 1.0 L/min with stroke volume 200 µL at 130 strokes/min. An expiratory tube was submerged in water to apply 2 cmH_2_O positive end‐expiratory pressure. Electrocardiogram (one lead) was recorded continuously until the end of the experiment with PowerLab/4SP system using LabChart 7 software (ADInstruments). An incision was made at the fourth intercostal space. Equal volumes of saline containing the indicated numbers of ExoDiff, ExoPr0 or PBS were injected via a jugular vein 5 min prior to heart ischaemia. The assignment of animals to groups was randomized, and the investigator was blinded to the treatments. Myocardial IR injury was induced by ligation of the left anterior descending artery with a silk suture for 40 min followed by 2 h reperfusion.^38^ Myocardial ischaemia was confirmed by changes in electrocardiogram and blanching of myocardium distal to the suture.

The hearts excised post‐reperfusion were cannulated and washed with saline. The previously blocked artery was re‐closed with the suture and the heart was perfused with 1% Evan's blue dye in saline (w/v) to determine the area at risk using standard methods.^38^ Consequently, hearts were frozen, sectioned and stained with 1% triphenyl tetrazolium chloride in phosphate buffer (w/v; pH7.4) at 37°C for 15 min. Heart sections were fixed with 4.0% formaldehyde solution in water (v/v) overnight. The sections were scanned with CanoScan LiDE 220 scanner (Cannon) and analysed. The area at risk (Evan's Blue‐negative), the infarct size (not stained with Evan's Blue and triphenyl tetrazolium chloride) and the non‐risk area (Evan's Blue‐positive) were determined using ImageJ (National Institutes of Health). The infarct size was measured as a percentage of the area at risk. Hearts with <30% risk area were excluded from analysis.

### 2.10 Cell cultures

HL‐1 cardiac muscle cells were grown in flasks coated with fibronectin and gelatine using supplemented Claycomb medium as previously described (Sigma‐Aldrich).^39^ Cells were incubated in humidified atmosphere (95% air, 5% CO_2_, 37°C).

### 2.11 Mitochondrial permeability transition pore opening assay

HL‐1 cells were seeded 100 000 cells per glass bottom dish (Greiner One, 627965). The following day, the sensitivity of the mitochondrial permeability transition pore to stress‐induced opening was assessed by using an oxidative stress model.^40^ Specifically, cells were incubated with 3 μmol/L tetramethylrhodamine (TMRM) in recording buffer for 15 min (ThermoFisher Scientific, T668). Due to its positive charge, TMRM is rapidly sequestered within mitochondria in a Nernstian fashion. At this dye concentration, the fluorescence signal is initially quenched, but mPTP opening causes the mitochondria to depolarize, and TMRM diffuses into the larger volume of the cytosol, in which it dequenches, becoming brighter. In HL‐1 cells, this results in a gradual increase in signal until maximal dye dequench. The known mPTP inhibitor, cyclosporine A, was used as a positive control. The recording buffer contained 156 mmol/L NaCl, 10 mmol/L HEPES, 10 mmol/L glucose, 3 mmol/L KCl, 2 mmol/L MgSO_4_.7H_2_O, 2 mmol/L CaCl_2_ and 1.25 mmol/L K_2_HPO_4_ in water (pH7.4). Cells were treated with 0.2 µM cyclosporin A (Cell Guidance Systems, SM43), DMSO, 10^9^ ‐ 10^10^ particles/mL ExoDiff or ExoPr0, 15 μmol/L LY294002, 50 μmol/L PD98059, 1 μmol/L SC144, 0.1 μmol/L Ruxolitinib or 5 μmol/L TAK242 (compounds are from Tocris unless otherwise indicated) during and after the 15 min TMRM loading period. Time‐lapse recordings of cells were made using a Leica TCS SP5 confocal microscope equipped with a 20× objective, 543 nm laser line of a Helium‐Neon laser for excitation (Leica Microsystems). The settings were as follows: 4 s time interval, 40% laser power and standard Leica TRITC emission settings. The fluorescence intensity at three separate locations per dish, each containing ~100 cells, was analysed using LAS AF software and the average intensity calculated. The half‐time taken for TMRM to maximally dequench was determined and used as an index of mPTP sensitivity. Using this assay, a longer time to mitochondrial depolarization indicates greater resistance of the mPTP to opening. The resultant value was considered as one replicate, and the experiment was repeated using different cell preparations.

### 2.12 Statistical analysis

Data are shown as mean ±SEM. For statistical analysis, one‐way ANOVA was followed by post‐test analysis by the Tukey test for multiple comparisons. *P* <.05 was considered significant. *P <.05; **P <.01, ***P <.001.

## 3 RESULTS

### 3.1 Exosome characterisation

**FIGURE 1:**
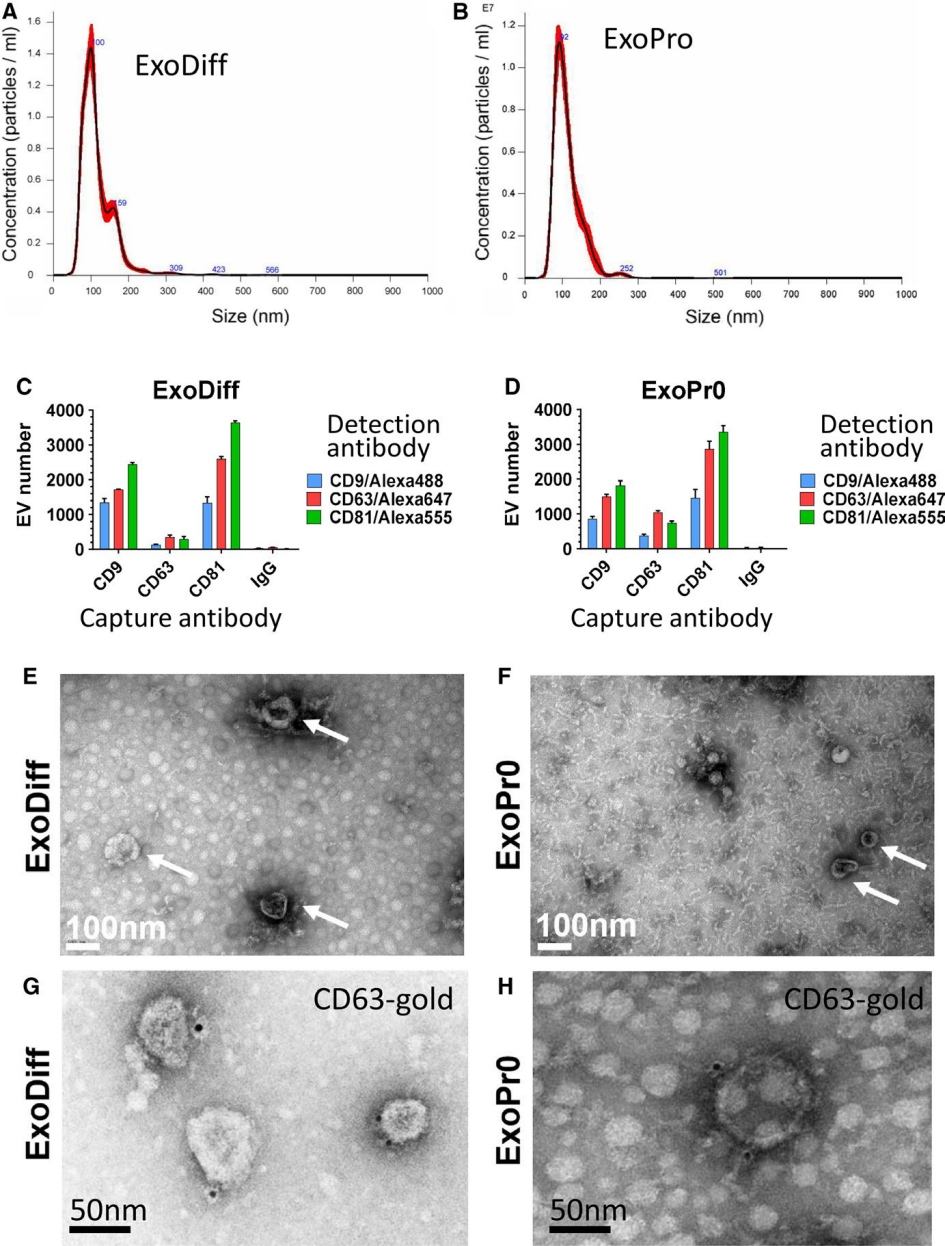
ExoDiff and ExoPr0 exosome characterisation. (A, B) Size distribution of ExoDiff and ExoPr0 samples were measured using nanoparticle tracking analysis. The data are shown as mean of three analyses with standard deviation in red. (C, D) The distribution of exosome marker expression (CD9, CD63 and CD81) on ExoDiff and ExoPr0, at the level of individuals’ exosomes, obtained using single particle interferometric reflectance imaging. Specific antibody or IgG negative control (X‐axis) was used to capture exosomes followed by detection with the fluorescent secondary antibody indicated. (E, F) Images of exosome samples were obtained using transmission electron microscopy. White arrows indicate cup‐shaped vesicles. The white bar indicates the scale (100 nm). (G,H) Immunolabelling of EVs with anti‐cd63 and gold‐tagged secondary antibody (block dots). Scale bar 50 nm

Exosomes were quantified and characterized both in the bulk sample and as single nanovesicles in accordance with the latest guidelines produced by the International Society for Extracellular Vesicles.^25^ Sample particle number and size were measured by nanoparticle tracking analysis. The particle size distribution of ExoDiff and ExoPr0 suggests that particles were predominantly within the exosome size range (Figure 1A, B). The modal particle size was similar in both samples (89.5 vs 87.3 nm in ExoDiff and ExoPr0). The particle concentration was ~4 times higher in ExoDiff compared with ExoPr0 (4.13 × 10^12^ vs 1.09 × 10^12^ particles/mL, respectively). To confirm the difference in exosome concentrations, protein concentration was determined with spectrophotometry. Indeed, ExoDiff contained more protein than ExoPr0 (490 vs 190 μg/mL, respectively). The particle per protein unit ratio, which is an alternative measure of exosome purity, was 8.5 × 10^9^ particles/µg for ExoDiff and 5.9 × 10^9^ particles/µg for ExoPr0.^41^ We also measured the ratio of protein to lipid content, as an alternative method of assessing purity^37^ and found that it was approximately two times greater in ExoDiff compared with ExoPr0 (33.3 ± 2.6 μg protein/μg lipid and 17.2 ± 4.9 μg protein/μg lipid, respectively).

Next, the presence of CD9, CD63 and CD81 in ExoDiff and ExoPr0 samples was confirmed with an ELISA‐based DELFIA assay. These tetraspanin family proteins have been validated as extracellular vesicle (EV) and exosome markers.^25^, ^42^ Positive signal for each of these tetraspanins was detected relative to control vehicle (Table 1). To verify the co‐localization of tetraspanins on the putative exosomes and compare their relative expression, single particle interferometric reflectance imaging (SP‐IRIS) was used to image EVs incubated with a mixture of fluorescent antibodies against the three tetraspanins.^35^, ^43^ This technique allows a comparison between relative levels of exosome marker proteins at the level of individual exosomes and is useful both as a ‘fingerprint’ of exosome type and subtypes within a sample. Both ExoDiff and ExoPr0 were found to contain exosomes that were double‐positive for the various tetraspanin combinations (Figure 1C, D). Non‐specific binding of EVs to mouse IgG was negligible. The relative expression ratios were similar between ExoDiff and ExoPr0 suggestive of similar exosome content in the samples. We confirmed the presence of ‘cup‐shaped’ EVs the size of exosomes in both ExoDiff and ExoPr0 samples by electron microscopy (Figure 1E, F). Finally, to further confirm the presence of CD63 on EVs, we performed electron microscopy imaging on EV samples immune‐labelled with an antibody recognizing CD63 (Figure 1G,H). Collectively, the characterization data suggest that both samples contained purified exosomes.

**TABLE 1:** The presence of CD9, CD63 and CD81 was confirmed in ExoDiff and ExoPr0 samples by DELFIA

Sample	Secondary antibody alone (background)	CD9	CD63	CD81
ExoDiff	2,639 ± 483	31,916 ± 3,942	104,477 ± 13,225	157,990 ± 18,255
ExoPro		35,035 ± 2,762	245,297 ± 19,033	273,792 ± 22,977

Note

The data are shown as mean signal of triplicates from one sample; arbitrary units.

### 3.2 ExoDiff are cardioprotective in a mouse ischaemia/reperfusion model

**FIGURE 2:**
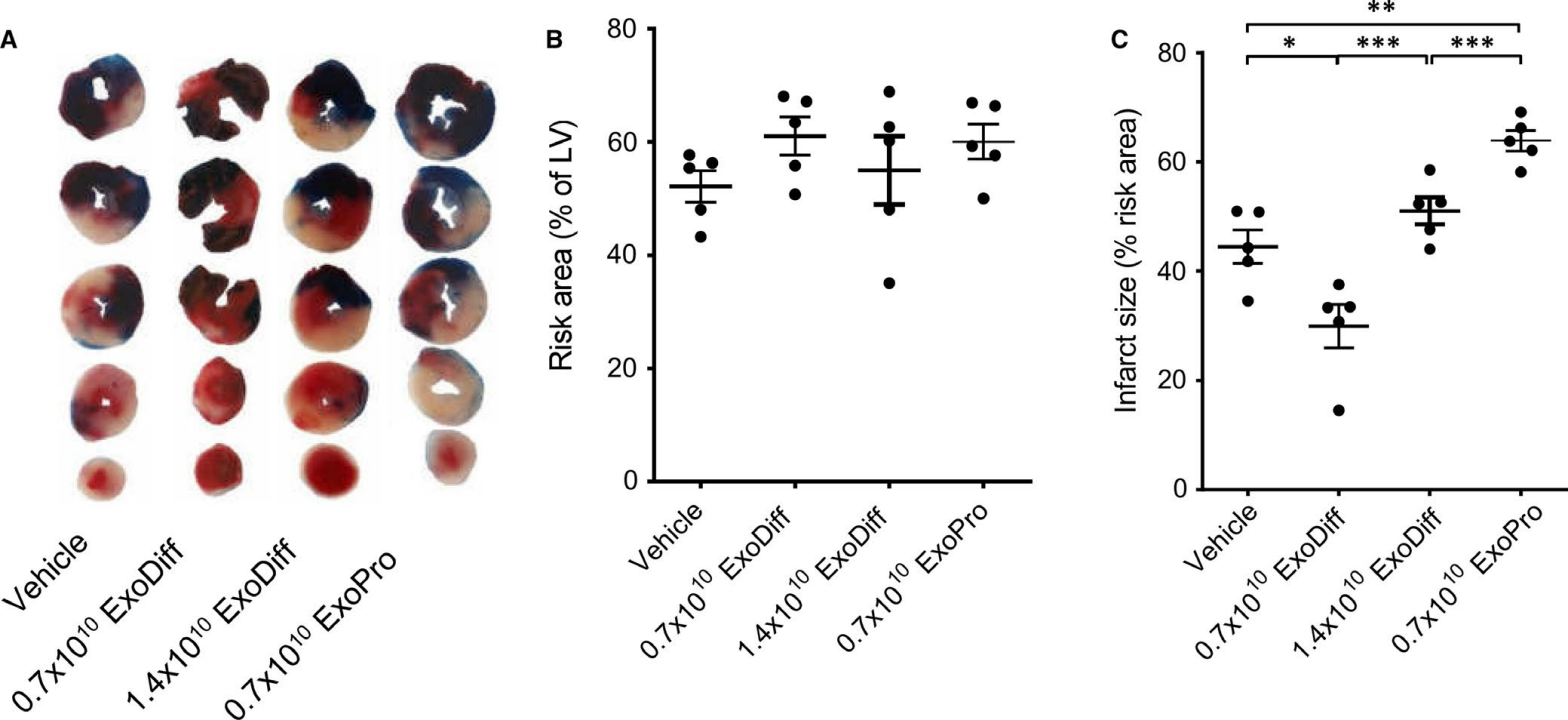
ExoDiff, and not ExoPr0, reduced infarct size in in vivo model of ischaemia/reperfusion injury. Wild‐type mice were injected intravenously with the indicated numbers of ExoDiff, ExoPr0 or vehicle (PBS), 5 min prior to regional myocardial ischaemia. (A) Following 40 min ligation of the left anterior descending artery and 2 h reperfusion, the hearts were excised, stained with Evan's Blue and TTC (representative slices are shown). (B) The ischaemic risk area, as a percentage of the left ventricle (LV) area, was similar between all groups. (C) Infarct size as a percentage risk area. The data are shown as mean ±SEM. Each dot represents one mouse heart. *P <.05; **P <.01; ***P <.001, between indicated groups

ExoDiff and ExoPr0 were investigated for their ability to limit infarct size in an in vivo model of cardiac IR injury. Wild‐type mice were injected with equal volumes of ExoDiff, ExoPr0 or vehicle (PBS) via the jugular vein, 5 min prior to regional myocardial ischaemia, which was achieved by ligation of the left anterior descending artery. After 40 min, the ligature was removed and the heart was reperfused. After 2 h reperfusion, the hearts were excised and the area at risk (AAR) and infarct size were determined (Figure 2A). The AAR was equivalent in all groups, demonstrating that the surgery was equivalent in each group (Figure 2B). Control mice, which were administered vehicle, had an infarct size of 44.5 ± 6.9% of the AAR (Figure 2C). The infarct size was significantly reduced to 29.9 ± 9.0% (*P* <.05) by the administration of 0.7 × 10^10^ particles of ExoDiff. Administration of 0.7 × 10^10^ particles ExoPr0 caused an increase in infarct size to 63.9 ± 4.1% (*P* <.01) (Figure 2C). Interestingly, when the concentration of ExoDiff administered was doubled, it no longer had any effect on infarct size (51.0 ± 5.1% infarct / AAR) (Figure 2C).

### 3.3 ExoDiff delays ROS‐mediated mPTP opening in cardiomyocytes by activating gp130/JAK pathway

**FIGURE 3:**
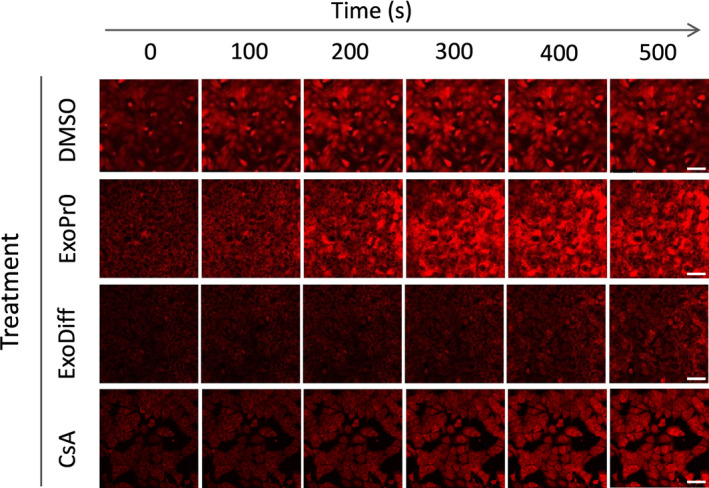
Representative images from the mPTP assay in HL‐1 cells. HL‐1 cardiomyocytes were pre‐loaded with a quenching concentration of TMRM, then subject to repeated confocal scanning with a HeNe laser. The ROS generated cause mPTP opening, mitochondrial depolarization and an increase in the fluorescent signal due to TMRM dequenching over time (horizontal). The top row shows the time to mPTP opening (ie increase in red fluorescence) in cells treated with DMSO vehicle control (DMSO). The time to mPTP opening is delayed by incubation with 10^10^ particles/mL ExoDiff or with 0.2 μmol/L cyclosporin A (CsA), but not 10^10^ particles/mL ExoPr0. Quantification is shown in Figure 4. Scale bars 100 μm

**FIGURE 4:**
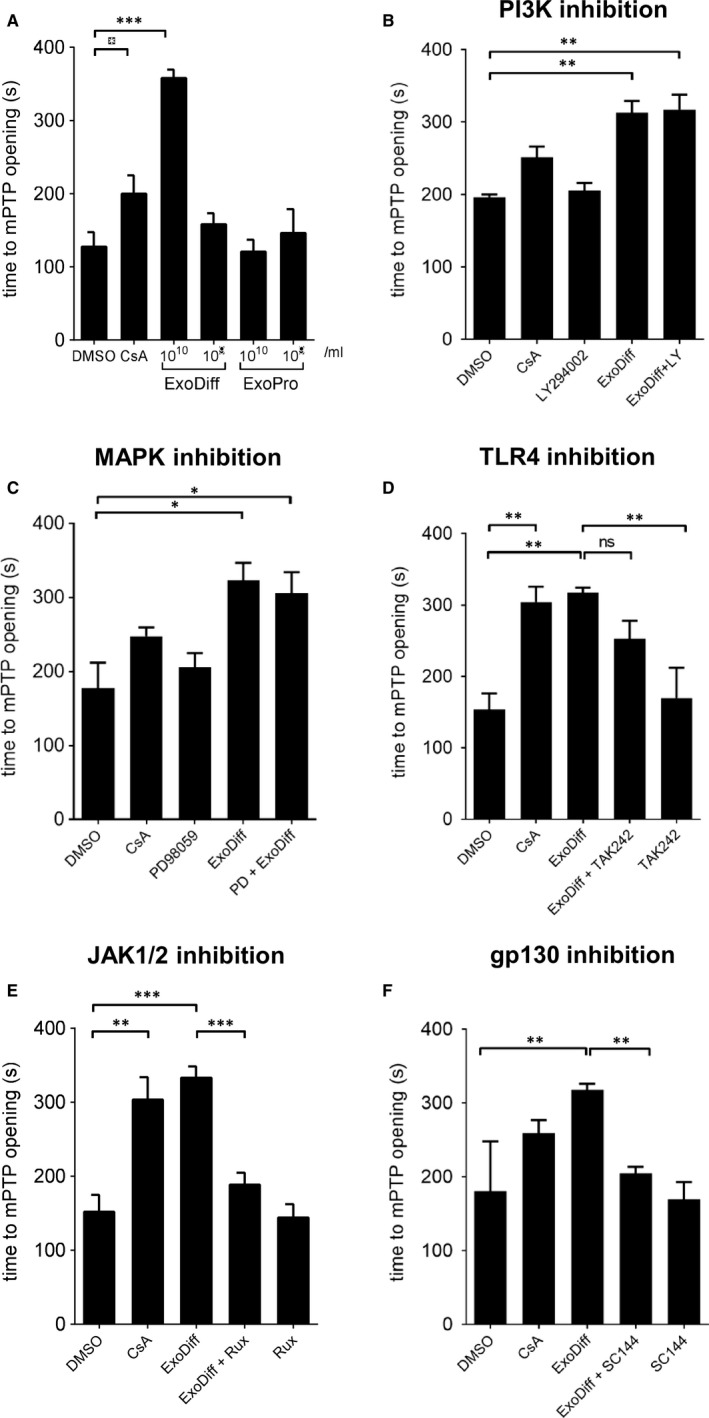
ExoDiff, and not ExoPr0, delays mPTP opening in cells via gp130 and JAK1/2. HL‐1 cells were treated with various treatments and inhibitors for 15 min prior to assessing the time until mPTP opening using the assay described in Figure 3. (A) HL‐1 cells were pre‐incubated with vehicle (DMSO), ExoDiff, ExoPr0, or 0.2 μmol/L cyclosporin A (CsA), (n = 6,6,3,3,3,2 in each group from left to right). (B) PI3K was inhibited with 15 μmol/L PI3K inhibitor LY294002 (LY), (n = 3,3,2,2,3). (C) MAPK was inhibited with 50 μmol/L MAPK inhibitor PD98059 (PD), (n = 3 in all groups). (D) TLR4 was inhibited with 5 μmol/L TAK242 (n = 5,6,5,4,4). (E) JAK1/2 was inhibited with 0.1 μmol/L Ruxolitinib (Rux), n= (3,3,4,4,2). (F) gp130 receptor was inhibited with 1 μmol/L SC144 (n = 7,6,5,5,5). Mean ±SEM is plotted. *P <.05; **P <.01; ***P <.001, as indicated

To elucidate the mechanism of cardioprotection conferred by ExoDiff, the effect of ExoDiff and ExoPr0 on mPTP opening was evaluated in vitro. Specifically, HL‐1 cardiac muscle cells were incubated with the fluorescent potentiometric mitochondrial dye, TMRM (Figure 3). TMRM sequesters in mitochondria in which it initiates singlet oxygen (ROS) production upon laser illumination.^40^ During and after TMRM incubation, cells were co‐incubated with exosomes, vehicle (DMSO) or the positive control of cyclosporin A (CsA), a known mPTP inhibitor. The sensitivity to mPTP opening was detected as an increase in fluorescent signal due to dequenching of the dye as it diffused out of the damaged mitochondria (Figure 3). As expected, mPTP opening was significantly delayed by CsA by more than 50% (Figure 4). Treatment with 10^10^ particles/mL ExoDiff delayed mPTP opening by more than 2.5‐fold (Figure 4A). A lower concentration of ExoDiff (10^9^ particles/mL) did not delay mPTP opening (Figure 4A). ExoPr0 had no effect at either concentration (Figure 4A). Therefore, ExoDiff, and not ExoPr0, may reduce the infarct size by protecting cardiomyocyte mitochondria from reactive oxygen species.

To discover how ExoDiff delays mPTP opening caused by ROS, HL‐1 cells were used as above, in the presence or absence of inhibitors of two RISK pathway kinases, MAPK and PI3K. Both inhibitors failed to reduce mPTP opening time in the presence of 10^10^ particles/mL ExoDiff (Figure 4B, C). Next, we used TAK242, an inhibitor of toll‐like receptor 4 (TLR4), the receptor which responds to damage‐associated molecular patterns (DAMPs). We have previously shown that TLR4 is involved in cardioprotection by plasma exosomes ^15^ (Figure 4D). However, TAK242 did not significantly affect the delay to mPTP opening caused by ExoDiff. Another major cardioprotective kinase pathway, referred to as the Survivor Activating Factor Enhancement (SAFE) pathway, involves JAK/STAT signalling.^44^ To investigate the role of the SAFE pathway in delaying mPTP opening in our assay, cells were treated with a specific inhibitor of JAK signalling called ruxolitinib.^45^ Ruxolitinib completely and significantly inhibited the delay to mPTP opening induced by ExoDiff (*P* <.001) (Figure 4E). Because JAK/STAT signalling is commonly activated by type I cytokines acting via the glycoprotein 130 (gp130) receptor, we also investigated a specific inhibitor of gp130, SC144.^46^ Inhibition of gp130 upstream with SC144 produced the same effect as inhibition of JAK, eliminating the protective effect of ExoDiff on mPTP opening (*P* <.01) (Figure 4F).

## 4 DISCUSSION

In this study, we showed that exosomes from differentiated neuronal stem cells can reduce infarct size induced by myocardial infarction. We also demonstrated that these exosomes delayed mPTP opening in cardiomyocytes, via a gp130/JAK/STAT pathway rather than the canonical RISK pathway (PI3K/AKT and ERK1/2). This extends previous studies that show exosomes from non‐cardiac cells can protect the heart ^13^, ^15^, ^16^, ^17^ and suggests that these GMP‐quality exosomes may be useful in limiting infarct size.

The purification method we used, of sequential TFF and SEC, was chosen to achieve optimal purity of exosomes.^41^ There is currently no consensus on the optimum way to determine exosome purity. According to the commonly used measure of exosome‐to‐protein ratio, in which a ratios > 3 × 10^10^ particles/μg is said to equate to high vesicular purity, the purity of ExoDiff and ExoPr0 is lower than for ultracentrifugation, which is unexpected. However, the concentration and size range of the particles detected by NTA and the presence of tetraspanin molecules demonstrates the presence of significant numbers of exosomes in our preparations. The presence of exosomes was further confirmed by electron microscopy, with and without immunostaining for CD63. The background staining seen by electron microscopy indicates that there may be some unidentified contaminants, particularly in ExoPr0. On the other hand, it has been reported that ultracentrifugation can damage vesicles and cause aggregation,^47^ so it possible that the vesicles obtained using TFF/SEC are less damaged than they would be if obtained by other techniques.

Here, we used exosomes derived from human cells in rodents. It is already well established that exosomes function across species, with human‐derived exosomes protective in mice.^48^, ^49^ This indicates that they act either via a specific mechanism that is highly conserved between species, or via a non‐specific mechanism. An example of the latter would be if exosomes behaved as ‘damage‐associated molecular patterns’ (DAMPs), stimulating the TLR receptors of the innate immune system. Indeed, we have previously shown that plasma‐derived exosomes protect the heart via TLR4 as protection was inhibited by TAK‐242.^15^ However, TAK‐242 had no effect in the current study, which suggests that this is not a general mechanism of exosomal cardioprotection. It also must be appreciated that, whereas we have demonstrated a differential effect between ExoDiff and ExoPr0 in an in vitro MPTP assay, we have not confirmed that this mechanism is involved in protection from ischaemia.

Interestingly, there was an opposite trend between the in vivo and in vitro results in terms of protection with ExoDiff. In vivo, only the lower dose (0.7 × 10^10^) and not the higher dose of ExoDiff (1.4 × 10^10^), showed a cardioprotective effect (Figure 3C). However, in vitro, treatment with 10^10^/mL but not 10^9^/mL ExoDiff significantly delayed opening of the mPTP (Figure 4A). It is difficult to make a direct comparison between the concentrations of exosomes used in for in vitro studies, and the quantity injected in vivo. We administered 0.7 × 10^10^ and 1.4 × 10^10^ ExoDiff in vivo, assuming an approximate blood volume of 1.4 mL in mice, as this equates to 0.5 × 10^10^ and 1.0 × 10^10^ ExoDiff per mL. However, it must be recognized that the effective plasma concentration achieved is unknown and is likely to be highly dependent on pharmacokinetics, albumin binding, cell receptor‐binding characteristics, etc Furthermore, compared with cardiomyocytes in vivo, the HL‐1 cardiomyocyte cell line used for in vitro studies may express different densities of the receptors that ExoDiff binds to and activates.

There is substantial interest in the miRNAs carried by exosomes and the role that they can have in recipient cells. However, in an acute study such as this, it is more likely that the effects seen are due to rapid ligand‐cell surface receptor interaction and intracellular signalling. Exosomes from various sources have been shown to activate the PI3K/AKT or MAPK/ERK1/2 components of the RISK pathway.^15^, ^18^, ^19^, ^20^ However, we found that the delay in mPTP opening caused by ExoDiff did not require the RISK pathway but did require the gp130/JAK pathway. Gp130 is associated with Janus kinases (JAKs): JAK1, JAK2 and TYK2. Upon gp130 activation, JAKs auto‐phosphorylate and activate STAT1, STAT3 and tyrosine phosphatase SHP‐2. Both JAK and STAT3 are kinases of a cardioprotective pathway known as the survivor activating factor enhancement (SAFE) pathway, which has previously been implicated in some cardioprotective strategies.^50^ Additionally, gp130 and SHP‐2 activate constituents of the RISK pathway, PI3K/AKT and MAPK/ERK pathways.^51^ Activation of either of SAFE or RISK pathways can result in inhibition of mPTP pore opening and improve cardiomyocyte survival.^6^, ^52^ Furthermore, there is crosstalk between these two cardioprotective pathways, such that if one is inhibited, the other is activated.^53^ Therefore, it seems that exosomes from different sources are able to activate different signalling pathways that can lead to cardioprotection—the SAFE pathway in the case of ExoDiff, and the RISK pathway in the case of some other exosomes. A limitation of our study is that we did not examine ROS production in vivo in mice and, therefore, cannot conclusively state that the reduction in ROS seen with ExoDiff treatment in vitro also occurs in vivo. In addition, we have not performed longer follow‐up experiments to assess the functional recovery of the heart post‐MI. Nevertheless, it would be interesting in the future to investigate whether combinations of exosomes from different sources would be more protective than those from a single source.^54^

Protection by ExoDiff required gp130 signalling and the downstream JAK/STAT pathway. A number of major cytokines can bind to gp130 including interleukin‐6 (IL‐6), leukaemia inhibitory factor (LIF), cardiotrophin‐1, ciliary neurotrophic factor, oncostatin M and interleukin‐11. This might suggest that ExoDiff exosomes display one or more of these cytokines on their surface. Alternatively, the IL‐6 receptor itself could be transferred on exosomes, as it has previously been shown to be shed from cells and found in circulating EVs.^55^ Interestingly, IL‐6 induces cardioprotection and preserves mitochondrial function in cardiomyocytes.^56^ However, we were unable to detect IL‐6 in conditioned medium using an ELISA (data not shown). Thus, the precise mechanism by which ExoDiff activate the gp130 pathway will require further investigation.

One important conclusion from our study is that exosomes from non‐cardiac stem cells also have the potential to be used to protect against myocardial infarction. Interestingly, though exosomes from a range of different sources have been reported to be cardioprotective, very few studies have reported a type of exosome not capable of protecting the heart. The one exception seems to be exosomes derived from dermal fibroblasts, which have been used as negative controls in some studies as they were not found to be protective.^19^ Certain conditions such as diabetes/hyperglycaemia can impair the exosome function.^23^, ^57^ On the other hand, exosomes isolated from cardiomyocytes cultured in conditions simulating ischaemia have been found to have an enhanced ability to promote cardiac angiogenesis.^58^ Although ExoDiff exosomes, which are isolated from differentiated cells, were cardioprotective in vivo and prevented mPTP opening in vitro, ExoPr0, which are isolated from proliferating cells, did not have these effects. The reason for this difference is not known, but in the future studies, we intend to examine their respective proteomes for clues that can explain this difference.

## CONFLICT OF INTEREST

RC is an employee of ReNeuron Ltd.

## AUTHOR CONTRIBUTION

**Miroslava Katsur:** Investigation (equal); Writing‐original draft (equal); Writing‐review & editing (equal). **Zhenhe He:** Investigation (equal). **Vladimir Vinokur:** Investigation (equal). **Randolph Corteling :** Resources (equal). **Derek Miles Yellon:** Conceptualization (equal); Project administration (equal); Supervision (equal); Writing‐review & editing (equal). **Sean Michael Davidson:** Conceptualization (equal); Data curation (equal); Funding acquisition (equal); Project administration (equal); Supervision (equal); Writing‐original draft (equal); Writing‐review & editing (equal).

## Data Availability

The data that support the findings of this study are available from the corresponding author upon reasonable request.

## References

[1] Thygesen K , Alpert JS , Jaffe AS , et al. Third universal definition of myocardial infarction. J Am Coll Cardiol. 2012;60:1581‐1598. 22958960 10.1016/j.jacc.2012.08.001

[2] Yellon DM , Hausenloy DJ . Myocardial reperfusion injury. N Engl J Med. 2007;357:1121‐1135. 17855673 10.1056/NEJMra071667

[3] Chouchani ET , Pell VR , Gaude E , et al. Ischaemic accumulation of succinate controls reperfusion injury through mitochondrial ROS. Nature. 2014;515:431‐435. 25383517 10.1038/nature13909 PMC4255242

[4] Davidson SM , Adameova A , Barile L , et al. Mitochondrial and mitochondrial‐independent pathways of myocardial cell death during ischaemia and reperfusion injury. J Cell Mol Med. 2020;24:3795‐3806. 32155321 10.1111/jcmm.15127 PMC7171390

[5] Halestrap AP , Davidson AM . Inhibition of Ca2(+)‐induced large‐amplitude swelling of liver and heart mitochondria by cyclosporin is probably caused by the inhibitor binding to mitochondrial‐matrix peptidyl‐prolyl cis‐trans isomerase and preventing it interacting with the adenine nucleotide translocase. Biochem J. 1990;268:153‐160. 2160810 10.1042/bj2680153 PMC1131405

[6] Davidson SM , Hausenloy D , Duchen MR , et al. Signalling via the reperfusion injury signalling kinase (RISK) pathway links closure of the mitochondrial permeability transition pore to cardioprotection. Int J Biochem Cell Biol. 2006;38:414‐419. 16280253 10.1016/j.biocel.2005.09.017

[7] Frias MA , Pedretti S , Hacking D , et al. HDL protects against ischemia reperfusion injury by preserving mitochondrial integrity. Atherosclerosis. 2013;228:110‐116. 23497785 10.1016/j.atherosclerosis.2013.02.003

[8] Schulman D , Latchman DS , Yellon DM . Urocortin protects the heart from reperfusion injury via upregulation of p42/p44 MAPK signaling pathway. Am J Physiol Heart Circ Physiol. 2002;283:H1481‐H1488. 12234800 10.1152/ajpheart.01089.2001

[9] Hausenloy DJ , Garcia‐Dorado D , Botker HE , et al. Novel targets and future strategies for acute cardioprotection: Position Paper of the European Society of Cardiology Working Group on Cellular Biology of the Heart. Cardiovasc Res. 2017;113:564‐585. 28453734 10.1093/cvr/cvx049

[10] Heusch G . Myocardial ischaemia‐reperfusion injury and cardioprotection in perspective. Nat Rev Cardiol. 2020;17:773‐789. 32620851 10.1038/s41569-020-0403-y

[11] Davidson SM , Yellon DM . Exosomes and cardioprotection ‐ A critical analysis. Mol Aspects Med. 2018;60:104‐114. 29122678 10.1016/j.mam.2017.11.004 PMC5861305

[12] Sluijter JPG , Davidson SM , Boulanger CM , et al. Extracellular vesicles in diagnostics and therapy of the ischaemic heart: Position paper from the working group on cellular biology of the heart of the european society of cardiology. Cardiovasc Res. 2018;114:19‐34. 29106545 10.1093/cvr/cvx211 PMC5852624

[13] Nie S , Wang X , Sivakumaran P , et al. Biologically active constituents of the secretome of human W8B2(+) cardiac stem cells. Sci Rep. 2018;8:1579. 29371689 10.1038/s41598-018-19855-4 PMC5785502

[14] Takov K , He Z , Johnston HE , et al. Small extracellular vesicles secreted from human amniotic fluid mesenchymal stromal cells possess cardioprotective and promigratory potential. Basic Res Cardiol. 2020;115:26. 32146560 10.1007/s00395-020-0785-3 PMC7060967

[15] Vicencio JM , Yellon DM , Sivaraman V , et al. Plasma exosomes protect the myocardium from ischemia‐reperfusion injury. J Am Coll Cardiol. 2015;65:1525‐1536. 25881934 10.1016/j.jacc.2015.02.026

[16] Liu H , Gao W , Yuan J , et al. Exosomes derived from dendritic cells improve cardiac function via activation of CD4(+) T lymphocytes after myocardial infarction. J Mol Cell Cardiol. 2016;91:123‐133. 26746143 10.1016/j.yjmcc.2015.12.028

[17] Figliolini F , Ranghino A , Grange C , et al. Extracellular vesicles from adipose stem cells prevent muscle damage and inflammation in a mouse model of hind limb ischemia: role of neuregulin‐1. Arterioscler Thromb Vasc Biol. 2020;40:239‐254. 31665908 10.1161/ATVBAHA.119.313506

[18] Sun XH , Wang X , Zhang Y , et al. Exosomes of bone‐marrow stromal cells inhibit cardiomyocyte apoptosis under ischemic and hypoxic conditions via miR‐486‐5p targeting the PTEN/PI3K/AKT signaling pathway. Thromb Res. 2019;177:23‐32. 30844685 10.1016/j.thromres.2019.02.002

[19] Barile L , Cervio E , Lionetti V , et al. Cardioprotection by cardiac progenitor cell‐secreted exosomes: role of pregnancy‐associated plasma protein‐A. Cardiovasc Res. 2018;114:992‐1005. 29518183 10.1093/cvr/cvy055

[20] Arslan F , Lai RC , Smeets MB , et al. Mesenchymal stem cell‐derived exosomes increase ATP levels, decrease oxidative stress and activate PI3K/Akt pathway to enhance myocardial viability and prevent adverse remodeling after myocardial ischemia/reperfusion injury. Stem Cell Res. 2013;10:301‐312. 23399448 10.1016/j.scr.2013.01.002

[21] Davidson SM , Andreadou I , Barile L , et al. Circulating blood cells and extracellular vesicles in acute cardioprotection. Cardiovasc Res. 2019;115:1156‐1166. 30590395 10.1093/cvr/cvy314 PMC6529916

[22] Davidson SM , Riquelme JA , Zheng Y , et al. Endothelial cells release cardioprotective exosomes that may contribute to ischaemic preconditioning. Sci Rep. 2018;8:15885. 30367147 10.1038/s41598-018-34357-z PMC6203728

[23] Wider J , Undyala VVR , Whittaker P , et al. Remote ischemic preconditioning fails to reduce infarct size in the Zucker fatty rat model of type‐2 diabetes: role of defective humoral communication. Basic Res Cardiol. 2018;113:16. 29524006 10.1007/s00395-018-0674-1 PMC6776086

[24] Kalladka D , Sinden J , Pollock K , et al. Human neural stem cells in patients with chronic ischaemic stroke (PISCES): a phase 1, first‐in‐man study. Lancet. 2016;388:787‐796. 27497862 10.1016/S0140-6736(16)30513-X

[25] Thery C , Witwer KW , Aikawa E , et al. Minimal information for studies of extracellular vesicles 2018 (MISEV2018): a position statement of the International Society for Extracellular Vesicles and update of the MISEV2014 guidelines. J Extracell Vesicles. 2018;7:1535750. 30637094 10.1080/20013078.2018.1535750 PMC6322352

[26] Sinden JD , Hicks C , Stroemer P , et al. Human neural stem cell therapy for chronic ischemic stroke: Charting progress from laboratory to patients. Stem Cells Dev. 2017;26:933‐947. 28446071 10.1089/scd.2017.0009 PMC5510676

[27] Muir KW , Bulters D , Willmot M , et al. Intracerebral implantation of human neural stem cells and motor recovery after stroke: multicentre prospective single‐arm study (PISCES‐2). J Neurol Neurosurg Psychiatry. 2020;91:396‐401. 32041820 10.1136/jnnp-2019-322515 PMC7147186

[28] Colao IL , Corteling R , Bracewell D , et al. Manufacturing exosomes: A promising therapeutic platform. Trends Mol Med. 2018;24:242‐256. 29449149 10.1016/j.molmed.2018.01.006

[29] Stevanato L , Thanabalasundaram L , Vysokov N , et al. Investigation of content, stoichiometry and transfer of miRNA from human neural stem cell line derived exosomes. PLoS One. 2016;11:e0146353. 26752061 10.1371/journal.pone.0146353 PMC4713432

[30] Thomas RJ , Hope AD , Hourd P , et al. Automated, serum‐free production of CTX0E03: a therapeutic clinical grade human neural stem cell line. Biotechnol Lett. 2009;31:1167‐1172. 19343502 10.1007/s10529-009-9989-1

[31] Pollock K , Stroemer P , Patel S , et al. A conditionally immortal clonal stem cell line from human cortical neuroepithelium for the treatment of ischemic stroke. Exp Neurol. 2006;199:143‐155. 16464451 10.1016/j.expneurol.2005.12.011

[32] Gardiner C , Ferreira YJ , Dragovic RA , et al. Extracellular vesicle sizing and enumeration by nanoparticle tracking analysis. J Extracell Vesicles. 2013;2:19671. 10.3402/jev.v2i0.19671 PMC3760643 24009893

[33] Trumbo TA , Schultz E , Borland MG , et al. Applied spectrophotometry: analysis of a biochemical mixture. Biochem Mol Biol Educ. 2013;41:242‐250. 23625877 10.1002/bmb.20694

[34] Allicotti G , Borras E , Pinilla C . A time‐resolved fluorescence immunoassay (DELFIA) increases the sensitivity of antigen‐driven cytokine detection. J Immunoassay Immunochem. 2003;24:345‐358. 14677653 10.1081/IAS-120025772

[35] Daaboul GG , Gagni P , Benussi L , et al. Digital detection of exosomes by interferometric imaging. Sci Rep. 2016;6:37246. 27853258 10.1038/srep37246 PMC5112555

[36] Théry C , Amigorena S , Raposo G , Clayton A . Isolation and characterization of exosomes from cell culture supernatants and biological fluids. Curr Protoc Cell Biol. 2006; Chapter 3: Unit 3 22. 10.1002/0471143030.cb0322s30 18228490

[37] Visnovitz T , Osteikoetxea X , Sodar BW , et al. An improved 96 well plate format lipid quantification assay for standardisation of experiments with extracellular vesicles. J Extracell Vesicles. 2019;8:1565263. 30728922 10.1080/20013078.2019.1565263 PMC6352952

[38] Botker HE , Hausenloy D , Andreadou I , et al. Practical guidelines for rigor and reproducibility in preclinical and clinical studies on cardioprotection. Basic Res Cardiol. 2018;113:39. 30120595 10.1007/s00395-018-0696-8 PMC6105267

[39] Claycomb WC , Lanson NA Jr , Stallworth BS , et al. HL‐1 cells: a cardiac muscle cell line that contracts and retains phenotypic characteristics of the adult cardiomyocyte. Proc Natl Acad Sci U S A. 1998;95:2979‐2984. 9501201 10.1073/pnas.95.6.2979 PMC19680

[40] Hausenloy DJ , Yellon DM , Mani‐Babu S , et al. Preconditioning protects by inhibiting the mitochondrial permeability transition. Am J Physiol Heart Circ Physiol. 2004;287:H841‐H849. 15072953 10.1152/ajpheart.00678.2003

[41] Webber J , Clayton A . How pure are your vesicles? J Extracell Vesicles. 2013;2. 10.3402/jev.v2i0.19861 PMC3760653 24009896

[42] Andreu Z , Yanez‐Mo M . Tetraspanins in extracellular vesicle formation and function. Front Immunol. 2014;5:442. 25278937 10.3389/fimmu.2014.00442 PMC4165315

[43] Tkach M , Kowal J , Thery C . Why the need and how to approach the functional diversity of extracellular vesicles. Philos Trans R Soc Lond B Biol Sci. 2018;373:20160479. 29158309 10.1098/rstb.2016.0479 PMC5717434

[44] Lecour S . Activation of the protective Survivor Activating Factor Enhancement (SAFE) pathway against reperfusion injury: Does it go beyond the RISK pathway? J Mol Cell Cardiol. 2009;47:32‐40. 19344728 10.1016/j.yjmcc.2009.03.019

[45] Quintas‐Cardama A , Vaddi K , Liu P , et al. Preclinical characterization of the selective JAK1/2 inhibitor INCB018424: therapeutic implications for the treatment of myeloproliferative neoplasms. Blood. 2010;115:3109‐3117. 20130243 10.1182/blood-2009-04-214957 PMC3953826

[46] Xu S , Grande F , Garofalo A , et al. Discovery of a novel orally active small‐molecule gp130 inhibitor for the treatment of ovarian cancer. Mol Cancer Ther. 2013;12:937‐949. 23536726 10.1158/1535-7163.MCT-12-1082

[47] Linares R , Tan S , Gounou C , et al. High‐speed centrifugation induces aggregation of extracellular vesicles. J Extracell Vesicles. 2015;4:29509. 26700615 10.3402/jev.v4.29509 PMC4689953

[48] Lai RC , Arslan F , Lee MM , et al. Exosome secreted by MSC reduces myocardial ischemia/reperfusion injury. Stem Cell Res. 2010;4:214‐222. 20138817 10.1016/j.scr.2009.12.003

[49] Liu J , Yellon DM , Davidson SM . Evaluating early and delayed cardioprotection by plasma exosomes in simulated ischaemia–reperfusion injury. Biosci Horiz. 2015;8:1‐11.

[50] Hadebe N , Cour M , Lecour S . The SAFE pathway for cardioprotection: is this a promising target? Basic Res Cardiol. 2018;113:9. 29335904 10.1007/s00395-018-0670-5

[51] Tvedt THA , Ersvaer E , Tveita AA , et al. Interleukin‐6 in Allogeneic Stem Cell Transplantation: Its Possible Importance for Immunoregulation and As a Therapeutic Target. Front Immunol. 2017;8:667. 28642760 10.3389/fimmu.2017.00667 PMC5462914

[52] Boengler K , Hilfiker‐Kleiner D , Heusch G , et al. Inhibition of permeability transition pore opening by mitochondrial STAT3 and its role in myocardial ischemia/reperfusion. Basic Res Cardiol. 2010;105:771‐785. 20960209 10.1007/s00395-010-0124-1 PMC2978889

[53] Somers SJ , Frias M , Lacerda L , et al. Interplay between SAFE and RISK pathways in sphingosine‐1‐phosphate‐induced cardioprotection. Cardiovasc Drugs Ther. 2012;26:227‐237. 22392184 10.1007/s10557-012-6376-2

[54] Davidson SM , Ferdinandy P , Andreadou I , et al. Multitarget Strategies to Reduce Myocardial Ischemia/Reperfusion Injury: JACC Review Topic of the Week. J Am Coll Cardiol. 2019;73:89‐99. 30621955 10.1016/j.jacc.2018.09.086

[55] Schumacher N , Meyer D , Mauermann A , et al. Shedding of Endogenous Interleukin‐6 Receptor (IL‐6R) Is Governed by A Disintegrin and Metalloproteinase (ADAM) Proteases while a Full‐length IL‐6R Isoform Localizes to Circulating Microvesicles. J Biol Chem. 2015;290:26059‐26071. 26359498 10.1074/jbc.M115.649509 PMC4646259

[56] Smart N , Mojet MH , Latchman DS , et al. IL‐6 induces PI 3‐kinase and nitric oxide‐dependent protection and preserves mitochondrial function in cardiomyocytes. Cardiovasc Res. 2006;69:164‐177. 16219301 10.1016/j.cardiores.2005.08.017

[57] Davidson SM , Riquelme JA , Takov K , et al. Cardioprotection mediated by exosomes is impaired in the setting of type II diabetes but can be rescued by the use of non‐diabetic exosomes in vitro. J Cell Mol Med. 2018;22:141‐151. 28840975 10.1111/jcmm.13302 PMC5742744

[58] Ribeiro‐Rodrigues TM , Laundos TL , Pereira‐Carvalho R , et al. Exosomes secreted by cardiomyocytes subjected to ischaemia promote cardiac angiogenesis. Cardiovasc Res. 2017;113:1338‐1350. 28859292 10.1093/cvr/cvx118

